# Identification of a Novel Astrovirus in Pinnipeds

**DOI:** 10.3389/fmicb.2022.845601

**Published:** 2022-05-04

**Authors:** Peijun Zhang, Haoxiang Su, Ruoyan Peng, Jasper Fuk-Woo Chan, Shijie Bai, Gaoyu Wang, Yi Huang, Xiaoyuan Hu, Jun Luo, Sisi Liu, Youyou Li, Liying Xue, Fan Yang, Mingming Zhao, Yun Zhang, Chuanning Tang, Shu Shen, Xiuji Cui, Lina Niu, Gang Lu, Kwok-Yung Yuen, Fei Deng, Weijia Zhang, Feifei Yin, Jiang Du

**Affiliations:** ^1^NHC Key Laboratory of Systems Biology of Pathogens, Institute of Pathogen Biology, Chinese Academy of Medical Sciences and Peking Union Medical College, Beijing, China; ^2^Marine Mammal and Marine Bioacoustics Laboratory, Institute of Deep-Sea Science and Engineering, Chinese Academy of Sciences, Sanya, China; ^3^Key Laboratory of Tropical Translational Medicine of Ministry of Education, Hainan Medical University, Haikou, China; ^4^Laboratory of Deep Sea Microbial Cell Biology, Institute of Deep-Sea Science and Engineering, Chinese Academy of Sciences, Sanya, China; ^5^Department of Pathogen Biology, Hainan Medical University, Haikou, China; ^6^Hainan Medical University-The University of Hong Kong Joint Laboratory of Tropical Infectious Diseases, Hainan Medical University, Haikou, China; ^7^State Key Laboratory of Emerging Infectious Diseases, The University of Hong Kong, Pokfulam, Hong Kong SAR, China; ^8^Laboratory of Marine Viruses and Molecular Biology, Institute of Deep-Sea Science and Engineering, Chinese Academy of Sciences, Sanya, China; ^9^Dalian Sunasia Tourism Holding Co., Ltd., Dalian, China; ^10^Qingdao Polar Haichang Ocean Park, Qingdao, China; ^11^TCM School of Hainan Medical University, Haikou, China; ^12^State Key Laboratory of Virology and National Virus Resource Center, Wuhan Institute of Virology, Chinese Academy of Sciences, Wuhan, China

**Keywords:** genetic diversity, novel astrovirus, pinnipeds, evolution analysis, recombination

## Abstract

Astroviruses infect human and animals and cause diarrhea, fever, and vomiting. In severe cases, these infections may be fatal in infants and juvenile animals. Previous evidence showed that humans in contact with infected animals can develop serological responses to astroviruses. Mamastrovirus 11 is a species of *Mamastrovirus* and was first reported in 2018. It was detected in the fecal samples of a California sea lion. The genome sequence of its capsid protein (CP) was submitted to GenBank. However, the genome sequence of its non-structural protein region was not elucidated. In the present study, we characterized the genome sequences of the novel astroviruses AstroV-HMU-1 and AstroV-like-HMU-2. These were obtained from California sea lions (*Zalophus californianus*) and walruses (*Odobenus rosmarus*) presenting with loose stools. A phylogenetic analysis revealed that the CP of AstroV-HMU-1 closely clustered with Mamastrovirus 11 while its RNA-dependent RNA polymerase (RdRp) and serine protease (SP) were closely related to the mink astrovirus in the genus *Mamastrovirus*. The genome of AstroV-HMU-1 provided basic information regarding the NS protein regions of Mamastrovirus 11. Recombination analyses showed that the genomes of *Z. californianus* AstroV-HMU-1, VA2/human and the mink astrovirus may have recombined long ago. The NS of AstroV-like-HMU-2 segregated from the Astroviridae in the deep root of the phylogenetic tree and exhibited 36% amino acid identity with other mamastroviruses. Thus, AstroV-like-HMU-2 was proposed as a member of a new genus in the unclassified Astroviridae. The present study suggested that that the loose stools of pinnipeds may be the result of occasional infection by this novel astrovirus. This discovery provides a scientific basis for future investigations into other animal-borne infectious diseases.

## Introduction

Astroviruses are leading causes of infectious diarrhea in children, the elderly, immunocompromised individuals, and a wide range of animals. The major clinical symptom is watery diarrhea. The diseases may be fatal in infants and juvenile animals (Boros et al., 2017; Vu et al., 2017). HAstV-1 was first detected in humans in 1975 (Appleton and Higgins, 1975). Since then, the reported incidence of astrovirus infection in humans and animals has increased. Astrovirus infections are ubiquitous and ∼90% of the human population aged >9 years presents with anti-HAstV-1 antibodies (Mitchell et al., 1999). High-throughput next-generation sequencing (NGS) methods have recently disclosed that astroviruses may be associated with aseptic encephalitis, meningitis, and meningoencephalomyelitis in humans and animals (Calistri and Palu, 2015; Cortez et al., 2019; Roach and Langlois, 2021).

Astroviruses are non-enveloped, single-stranded, positive-sense RNA viruses of the Astroviridae. *Mamastrovirus* includes 19 species while *Avastrovirus* includes three species. Four *Mamastrovirus* species are known to infect humans while the other 15 only infect mammals. The prototype strain is Mamastrovirus 1 (MAstV1) which corresponds to human astrovirus genotypes 1–8 (HAstV 1–8). Mamastrovirus 6 contains the human astrovirus clades Melbourne 1–3 (MLB1–3) (Finkbeiner et al., 2008). Mamastrovirus 8 (MAstV8) and Mamastrovirus 9 (MastV9) include the human astrovirus clades Virginia 2 and 4 (VA2, 4) and Virginia 1 and 3 (VA1, 3) (Finkbeiner et al., 2009), respectively. Astrovirus genomes are 6.8–7 kb long and contain three overlapping open reading frames (ORF1a, ORF1b, and ORF2). ORF1a and ORF1b encode a viral protease and a polymerase, respectively. ORF2 is expressed by a subgenomic RNA and encodes a VP90 capsid precursor protein. The 5′-terminus is linked to a viral protein genome-linked (VPg) protein while the 3′-terminus contains a poly (A) tract.

Humans in contact with turkeys may present with serological responses to turkey astrovirus (Meliopoulos et al., 2014). Furthermore, astrovirus strains previously limited to humans have also been detected in non-human primates (Karlsson et al., 2015). Hence, astroviruses may be able to cross species barriers (Johnson et al., 2017). Recent research has focused on invertebrates and vertebrates in ocean ecosystems to study the origins, evolution, and emergence of terrestrial viruses. Astroviruses in marine mammal hosts including California sea lions (*Zalophus californianus*), Stellar sea lions (*Eumetopias jubatus*), and common bottlenose dolphins (*Tursiops truncatus*) were first reported in 2009 (Rivera et al., 2010). Bayesian and maximum likelihood (ML) phylogenetic methods were recently used to demonstrate that the novel flaviviruses detected in crustaceans are more closely related to terrestrial vector-borne than classical insect-specific flaviviruses (Parry and Asgari, 2019). Therefore, the astroviruses in marine mammals may be closely related to those in terrestrial animals according to phylogenetic analysis. The discovery of new marine mammal viruses is significant for viral phylogenetic analysis and taxonomic research. Here, we used NGS and discovered two novel astroviruses in oral and anal swab samples of walruses and California sea lions. It was established that AstroV-like-HMU-2 is phylogenetically distinct from all other known astroviruses, and the nearly complete genome sequence of AstroV-HMU-1 was determined and characterized.

## Materials and Methods

### Pinniped Swab Samples

Between January and December 2018, 32 oral, nasal, and anal swabs were collected from six walruses (*Odobenus rosmarus*) and six California sea lions (*Z. californianus*) presenting with loose stools and housed at the aquaria of Qingdao Polar Haichang Ocean Park and Dalian Sunasia Tourism Holding Co., Ltd., China. The samples were immersed in maintenance medium in virus-sampling tubes (Yocon, Beijing, China), transported on ice to the laboratory within 24 h, and stored at −80°C.

### Ethical Animal Treatment Statement

All animals were treated according to the guidelines of the Regulations for the Administration of Laboratory Animals (Decree No. 2 of the State Science and Technology Commission of the People’s Republic of China, 1988). The sampling procedures were approved by the Ethics Committee of Hainan Medical University (Approval No. HMUEC20180059).

### Viral Nucleic Acid Library Construction and Next-Generation Sequencing

All 32 samples were combined by species into pool 30 for *O. rosmarus* and pool 35 for *Z. californianus*. The samples were passed through 0.45-μm filters. The filtrates were digested with DNase (Applied Biosystems, Santa Clara, CA, United States) and RNase One (Promega, Madison, WI, United States) to remove any unprotected nucleic acids. Total RNA was extracted with a QIAamp Viral RNA Mini Kit (Qiagen, Hilden, Germany, United States) according to the manufacturer’s instructions. The cDNA was generated with Superscript III Reverse Transcriptase (Invitrogen, Carlsbad, CA, United States) as previously described (Wu et al., 2018). The amplified viral nucleic acid libraries were analyzed with an Illumina HiSeq 2500 sequencer (Illumina, San Diego, CA, United States). The sequence data were deposited to the National Center for Biotechnology Information (NCBI; Bethesda, MD, United States) sequence reads archive under accession No. PRJNA650224. The raw sequence reads were filtered and valid sequences were obtained using previously described criteria (Yang et al., 2011).

### Taxonomic Assignment

Sequence similarity-based taxonomic assignments were conducted as previously described (Yang et al., 2011). Briefly, the viral origin of each read was determined *via* alignments with the NCBI non-redundant nucleotide (NT) and protein (NR) databases using BLASTn and BLASTx (−E: expected value < 10^–5^; −F: filter query sequence; default = T). The taxonomies of the aligned reads from all lanes with the highest BLAST scores (*E*-value < 10^––5^) were parsed and exported with a MEGAN6 MetaGenome Analyzer^1^ (Tamura et al., 2013).

### Astrovirus Genome Sequencing

Molecular clues from the metagenomic analyses were used to classify the sequence reads into virus families or genera with MEGA 6.^2^ Representative reads for the novel astrovirus were selected for genome sequencing and used in read-based PCR. Reads with accurate genomic locations were used to design specific nested PCR primers and identify partial genomes. PCR was performed with 2× Taq PCR Mastermix (Tiangen Biotech Co., Ltd., Beijing, China). Two microliters first-round PCR product was used as the template for the second PCR round. The thermal cycling conditions for PCR were 94°C for 5 min followed by 35 cycles of 94°C for 30 s, 52°C for 30 s, 72°C for 45 s, and a final elongation step at 72°C for 10 min. Pool samples 30 and 35 were selected for sequencing. The remaining genomic sequences were analyzed by genome walking and 5′- and 3′-rapid amplification of cDNA ends (RACE; Invitrogen, Carlsbad, CA, United States; TaKaRa Bio Inc., Kusatsu, Shiga, Japan). All primer sequences were based on the newly obtained reads and amplified sequences. The primers used are listed in Supplementary Tables 1, 2).

### Prevalence of Astrovirus Infection Among Pinnipeds

Viral RNA was isolated with a QIAamp Viral RNA Mini Kit (Qiagen, Hilden, Germany) using individual samples. The cDNA was generated using random primers and Superscript III Reverse Transcriptase (Invitrogen). The nearly complete genomic sequences of the viruses obtained by end amplification were used to design specific nested primers targeting the non-structural gene for PCR and screen for astroviruses in the oral, nasal, and anal swabs of walruses and California sea lions (Supplementary Table 3). PCR was performed using 2× Taq PCR Mastermix (Tiangen Biotech Co., Ltd., Beijing, China). Two microliters first-round PCR product was used as the template for the second PCR round. The thermal cycling conditions were 94°C for 5 min followed by 35 cycles of 94°C for 30 s, 57°C for 30 s, 72°C for 45 s, and a final elongation step at 72°C for 10 min. The PCR products were analyzed by 1.5% agarose gel electrophoresis and ultraviolet imaging.

### Genome Annotation

The nucleotide sequences of the genomes and the amino acid sequences of the open reading frames (ORFs) were deduced by comparing the sequences against those of other astroviruses. The conserved protein families and domains were predicted with Pfam,^3^ Blastp,^4^ and InterProScan 5 (see text footnote 3). Routine sequence alignments were performed with Clustal Omega.^5^

### Phylogenetic and Recombination Analysis

MEGA6.0 (see text footnote 2), the MUSCLE package,^6^ and their default parameters were used to align the nucleotide sequences and deduce the amino acid sequences. A phylogenetic tree was constructed by the ML method. The substitution model rtREV and the Freqs (+F) model were run using the model selection function in MEGA6.0 and 1,000 bootstrap replicates. Pairwise amino acid alignment between the novel pinniped astrovirus and the other Astroviridae was performed using the NCBI Basic Local Alignment Search Tool (BLAST).^7^ The alignments were analyzed with SimPlot v. 3.5.1^8^ using a sliding window of 1,000 nucleotides (nt) in 100-nt steps. Each strain was a query for each run.

## Results

### Discovery of a Novel Pinniped Astrovirus Through Next-Generation Sequencing

**FIGURE 1:**
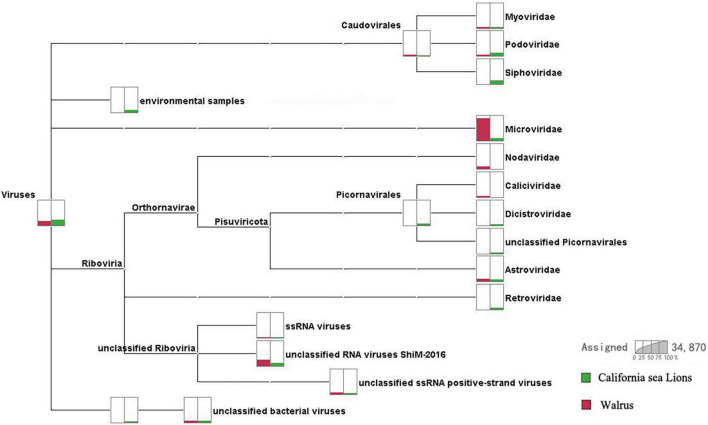
Comparison of virome compositions of walruses and California sea lions. The proportions of viral reads associated with California sea lions and walruses are highlighted in green and red, respectively.

We obtained 3.6 Gb of nucleotide data (34,870,258 valid reads, 150 bp long) from two pools according to species. Reads from archaea, bacteria, microbial eukaryotes such as fungi, and those with no significant similarities to the amino acid sequences in the non-redundant (NR) proteins database were removed. Pool 30 of *O. rosmarus* with 124,219 reads and pool 35 of *Z. californianus* with 5,026 reads most closely matched viral proteins from the NR database (∼0.37% of the total). A comparison of the virome composition between walrus and California sea lions is shown in Figure 1. We focused mainly on the mammalian viruses within these families. After excluding phages and insect, fungal, plant, and unclassified viruses, the proportion of reads corresponding to astroviruses accounted for 40.11 and 43.64% of the total mammalian viruses in pools 30 and 35, respectively.

### Genome Structure

**FIGURE 2:**
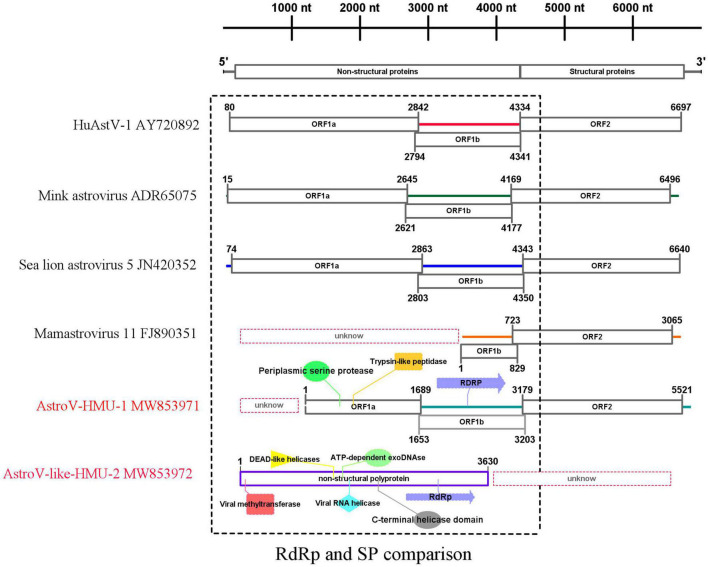
Genome organization and comparison of related astroviruses. Novel astroviruses discovered in the present study are highlighted in red.

We obtained a nearly complete genome sequence for the novel California sea lion astrovirus AstroV-HMU-1. We also obtained a complete coding sequence for the non-structural protein of the novel walrus virus AstroV-like-HMU-2 (GenBank accession Nos. MW853971–MW853972). The genome organization of AstroV-HMU-1 was characteristic of a mammalian astrovirus and comparable to those of related mammalian astroviruses (Figure 2). The genome sequence of AstroV-HMU-1 was 5,641 bp long and had 49.6% CG content. The BLAST similarity search revealed reading frame shifts in the translation around 1,642 and 3,205 nt. The capsid protein (CP) consisted of 781 amino acids. The non-structural protein consisted of a viral protease (SP) and a polymerase (Rdrp) which are typical of the astrovirus structure. The partial putative SP was 563 amino acids long and the putative RdRp was 517 amino acids long. The genome identity analysis showed that the CP of AstroV-HMU-1 had 96.71% identity to that of Mamastrovirus 11 (FJ890351) and <63.26% identity with other Mamastroviruses. The partial putative SP of AstroV-HMU-1 shared 58.11% identity with the SP of mink astrovirus (ADR65075) and had <54.20% identity with other Mamastroviruses. The putative RdRp of AstroV-HMU-1 shared 77.50% identity with the RdRp of mink astrovirus (ADR65075) and had <65.03% identity with other Mamastroviruses. However, there were relatively few reads corresponding to the astroviruses of Pool 35. The reads may only be related to the non-structural polyprotein region. We obtained the non-structural polyprotein sequence of AstroV-like-HMU-2 by reads-only PCR, designed specific gene primers, and used the 5′-RACE and 3′-RACE methods to amplify the remaining genome sequence. The amplification results were negative. Hence, we hypothesized that this novel astrovirus may lack poly(A)-tails. We used a Poly(A) Polymerase Kit (TaKaRa Bio Inc., Kusatsu, Shiga, Japan) to add poly(A)-tails to the 3′-hydroxyl end of the viral RNA. We then used the 3′-RACE method to re-amplify the 3′ ends. However, the amplification results remained negative. The non-structural polyprotein of AstroV-like-HMU-2 was 1,247 amino acids long and shared only 36.47% identity with those of bat astrovirus and <36% identity with other mamastroviruses.

For the putative non-structural protein, the conserved domains of AstroV-HMU-1 detected by Pfam were trypsin-like peptidase domain (148–268), periplasmic serine protease (123–279), and RNA-dependent RNA polymerase (625–979). The conserved domains of AstroV-like-HMU-2 detected by Pfam were viral methyltransferase (24–222) (involved in mRNA capping), viral RNA helicase (476–726), *C*-terminal helicase domain (674–726), ATP-dependent exoDNAse (473–739), DEAD-like helicases (456–575), and RdRp (942–1,115). The conserved domains of serine protease and RdRp were typical of astroviruses according to the NS structure but were absent in the NS sequence of AstroV-like-HMU-2. The conserved domains of RdRp were present in both AstroV-HMU-1 and AstroV-like-HMU-2 (Figure 2).

### Phylogenetic Analysis and Recombination

**FIGURE 3:**
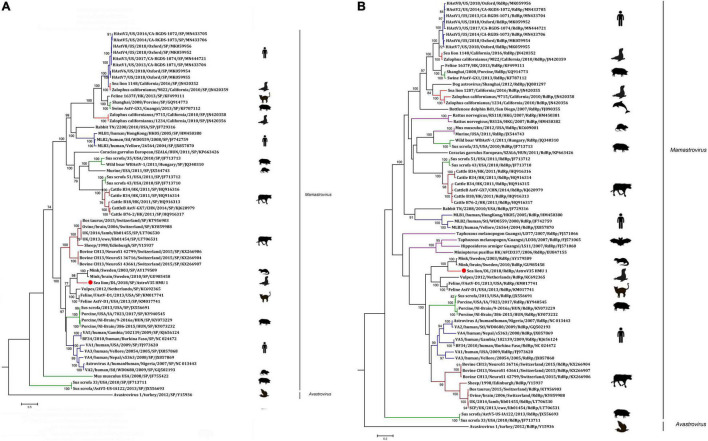
**(A)** Phylogenetic analyses of SP regions of AstroV-HMU-1 with reference sequences. **(B)** Phylogenetic analyses of RdRp regions of AstroV-HMU-1 with reference sequences. Maximum likelihood (ML) mtREV with Freqs (+F) model and γ-distributed with invariant sites (G + I) with 1,000 bootstrap replicates performed in MEGA6. Novel astrovirus is indicated by red circles (•). Bootstrap values are shown on branches.

**FIGURE 4:**
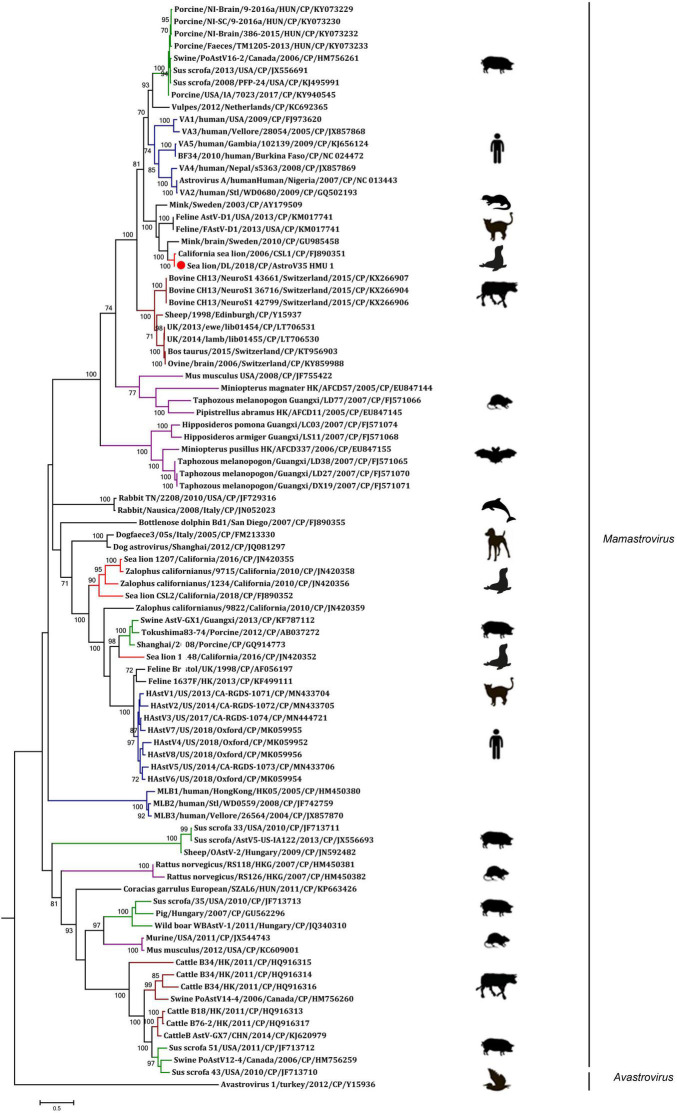
Phylogenetic analyses of CP regions of AstroV-HMU-1 with reference sequences. Maximum likelihood (ML) mtREV with Freqs (+F) model and γ-distributed with invariant sites (G + I) with 1,000 bootstrap replicates performed in MEGA6. Novel astrovirus is indicated by red circles (•). Bootstrap values are shown on branches.

**FIGURE 5:**
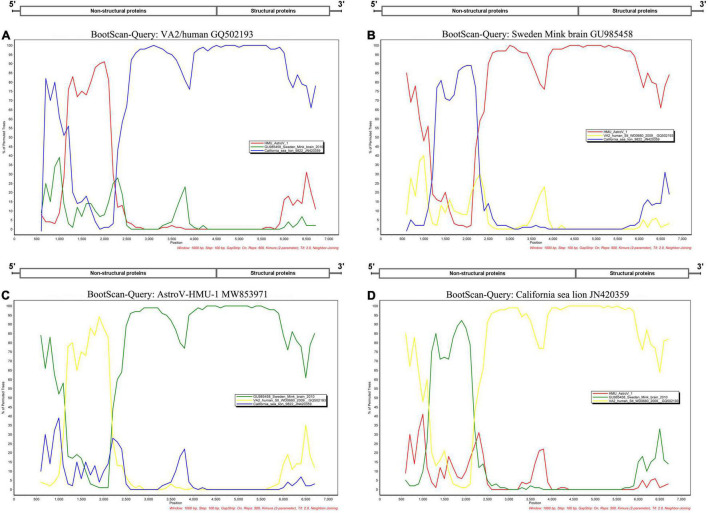
Bootscan analyses based on nearly full-length genomes of AstroV-HMU-1 and Swedish mink brain (GU985458), human VA2/human (GQ502193), and Californian sea lion (JN420359) strains. **(A)** VA2/human strain used as query sequence in analyses. **(B)** Swedish mink brain strain used as query sequence in analyses. **(C)** AstroV-HMU-1 strain used as query sequence in analyses. **(D)** Californian sea lion strains used as query sequence in analyses. Sliding window of 1,000 nt moving in 100-nucleotide steps.

A phylogenetic analysis of AstroV-HMU-1 was conducted using 89, 82, and 65 reference CP, RdRp, and SP sequences, respectively, from the Astroviridae in GenBank (accessed August 1, 2021). Evolutionary trees were constructed for the complete protein sequences of SP, RdRp, and CP (Figures 3, 4). The tree topology showed that the CP of AstroV-HMU-1 clustered with other *Z. californianus* astroviruses among the Mamastrovirus 11 species in genus *Mamastrovirus*. The tree topology data showed that the RdRp and SP of AstroV-HMU-1 clustered with mink astroviruses in the same branch and comprised a lineage distinct from other sea lion astroviruses. The recombination analyses (Figure 5) showed that the AstroV-HMU-1, SMS-AstV, and VA2/human viruses underwent substantial recombination events in the NS region.

**FIGURE 6:**
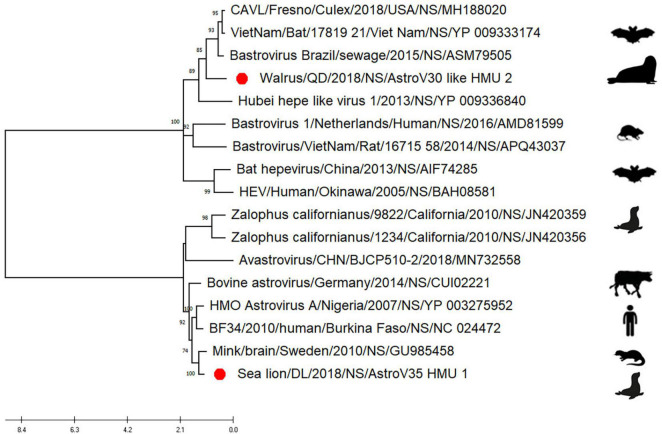
Phylogenetic analyses of NS regions of AstroV-like-HMU-2. Maximum likelihood (ML) mtREV with Freqs (+F) model and γ-distributed with invariant sites (G + I) with 1,000 bootstrap replicates performed in MEGA6. Novel astrovirus is indicated by red circles (•).

Phylogenetic analyses of AstroV-like-HMU-2 and AstroV-HMU-1 were conducted using the 16 NS in the positive-sense ssRNA virus related to bastroviruses (Sadeghi et al., 2018). The NS of AstroV-like-HMU-2 was segregated from the Astroviridae in the deep root of the phylogenetic tree (Figure 6) and formed an independent branch with Brazil/sewage, Bastrovirus-like_virus/VietNam/Bat/17819_21, and CAVL/Fresno. AstroV-HMU-1 was closely related to the mink astrovirus. This finding was consistent with results of the phylogenetic analyses of the SP and Rdrp regions.

### Prevalence of Astrovirus Infection Among Pinnipeds

Based on the genomic sequences of the novel astroviruses, gene-specific nested primers targeting the non-structural region for PCR were designed to screen for novel astroviruses in the mouths, noses, and anuses of pinnipeds. Two oral and three anal swabs of the California sea lions and one anal swab of the walruses were positive for the novel astrovirus. There was 0% positivity (0/4) for the nasal swabs, 14.3% positivity (2/14) for the oral swabs, and 28.6% (4/14) for the anal swabs (Table 1). The positive samples were identified from 939 bp of amplified products for AstroV-HMU-1 and 845 bp of amplified products for AstroV-like-HMU-2.

**TABLE 1:** Prevalence of AstroV-HMU-1, AstroV-like-HMU-2 in samples of captive walrus, California sea lions from Qingdao and Dalian Ocean Park.

Numbers	Animal species	Nose swab	Oral swab	Anal swab	Related viruses	Sampling date	Sampling place
23	*Odobenus rosmarus*	−	−	−		16.1.2018	Qingdao
24	*Odobenus rosmarus*	−	−	−		16.1.2018	Qingdao
23	*Odobenus rosmarus*	−	−	−		19.4.2018	Qingdao
24	*Odobenus rosmarus*	−	−	+	AstroV-like-HMU-2	19.4.2018	Qingdao
39	*Zalophus californianus*	Null	−	−		18.4.2018	Dalian
40	*Zalophus californianus*	Null	+	−	AstroV-HMU-1	18.4.2018	Dalian
41	*Zalophus californianus*	Null	Null	+	AstroV-HMU-1	18.4.2018	Dalian
42	*Zalophus californianus*	Null	Null	−		18.4.2018	Dalian
43	*Zalophus californianus*	Null	+	Null	AstroV-HMU-1	18.4.2018	Dalian
44	*Zalophus californianus*	Null	−	Null		18.4.2018	Dalian
45	*Zalophus californianus*	Null	−	−		18.4.2018	Dalian
39	*Zalophus californianus*	Null	Null	−		10.7.2018	Dalian
40	*Zalophus californianus*	Null	Null	−		10.7.2018	Dalian
41	*Zalophus californianus*	Null	−	+	AstroV-HMU-1	10.7.2018	Dalian
42	*Zalophus californianus*	Null	−	−		10.7.2018	Dalian
43	*Zalophus californianus*	Null	−	Null		10.7.2018	Dalian
44	*Zalophus californianus*	Null	−	Null		10.7.2018	Dalian
45	*Zalophus californianus*	Null	−	+	AstroV-HMU-1	10.7.2018	Dalian

*Samples found to be positive or negative by nested-PCR are indicated by + or − signs, respectively. Null indicates that the sample was not collected.*

## Discussion

The virome was composed of double-stranded (ds) DNA, dsRNA, retro-transcribing, single-stranded (ss) DNA, and ssRNA viruses and differed between walruses and California sea lions. The virus-associated reads of the California sea lion samples were either assigned to the families Myoviridae, Podoviridae, Siphoviridae, Microviridae, Dicistroviridae, Picornaviridae, Astroviridae, or Retroviridae or were unclassified. The virus-associated reads of the walrus samples were assigned to the families Myoviridae, Podoviridae, Microviridae, Caliciviridae, Dicistroviridae, Astroviridae or unclassified Riboviria viruses. Caliciviridae, Astroviridae, and Picornaviridae include numerous important mammalian viruses. Astrovirus-associated reads were detected in both the walrus and California sea lion samples. The positive rates of astrovirus infection determined for the individual samples reflected the possibility of a high incidence of astrovirus infection in pinnipeds.

The phylogenetic analysis of AstroV-HMU-1 was consistent with the genomic amino acid identity and recombination analyses and showed that the CP was assigned to Mamastrovirus 11 (California sea lion astroviruses) while the RdRp and SP clustered with the mink astroviruses. Hence, the genomes of AstroV-HMU-1, California sea lion 9,822, VA2/human, and mink astrovirus might have recombined long ago and later independently evolved into their natural hosts. Furthermore, Mamastrovirus 11 (FJ890351) is not associated with any genomic information for the NS protein regions in GenBank. Thus, *Z. californianus* AstroV-HMU-1 was proposed as a new member of the Mamastrovirus 11 species and provided genomic information for the NS protein regions.

The incidence of AstroV-like-HMU-2 was sporadic in samples from captive animals. AstroV-like-HMU-2 was detected in a single anal swab from *O. rosmarus*. However, the oral and nasal swab samples of this animal were negative. The oral and nasal swabs may not have collected sufficient virus-infected exfoliated host cells. Moreover, AstroV-like-HMU-2 only infected intestinal epithelial cells. The incidence of AstroV-like-HMU-1 was also sporadic. It was detected in only two oral and three anal swabs from *Z. californianus*. On April 18 and July 10, 2018, both anal swabs for *Z. californianus* No. 41 were positive. Hence, this individual might have been persistently infected with AstroV-HMU-1. Both oral swabs were also positive for AstroV-HMU-1. The route of astrovirus transmission may be fecal-oral. The pathogen could also be food- or water-borne (Walter and Mitchell, 2000). However, both corresponding anal swabs were negative for AstroV-HMU-1. In general, the positive rate for AstroV-HMU-1 was relatively low in *Z. californianus* even when positive oral or anal swabs were obtained. There was no direct evidence that AstroV-HMU-1 was associated with diarrhea, but it was nonetheless a possibility as California sea lions normally produce strip-like feces rather than loose stools.

According to the ICTV (Monroe et al., 2005), walruses are hosts of AstroV-like-HMU-2. Furthermore, the non-structural polyprotein of AstroV-like-HMU-2 shared <36.47% identity with those of other astroviruses. The NS of AstroV-like-HMU-2 segregated from the Astroviridae in the deep root of the phylogenetic tree. We propose that AstroV-like-HMU-2 is a novel species among the unclassified astroviruses. However, the astroviruses were classified on the basis of ORF2. Complete genome sequence information is required to determine whether AstroV-like-HMU-2 is, in fact, a new species. Future research should continue to elucidate the genomes of these viruses. Current astrovirus classification criteria do not accommodate the categorization of novel astroviruses. The Astroviridae may also include other genera besides *Mamastrovirus* and *Avastrovirus*. To confirm this theory, complete genome sequences of novel astroviruses should be uploaded to GenBank.

The novel pinniped astrovirus discovered and characterized here is a source of new genomic information that expands virological classification and provides a scientific basis for further investigations into animal-borne viral diseases.

## Data Availability Statement

The original contributions presented in the study are publicly available. This data can be found here: https://www.ncbi.nlm.nih.gov/bioproject/PRJNA650224.

## Ethics Statement

The animal study was reviewed and approved by the Ethics Committee of the Hainan Medical University.

## Author Contributions

JD, JF-WC, PZ, and K-YY designed the study. PZ, HS, GW, YH, SB, JL, SL, RP, XH, WZ, FY, XC, LN, YZ, YL, LX, CT, and GL collected the specimens and performed the experiments. JD, FFY, WZ, FD, and MZ analyzed the data. JD and FFY wrote the manuscript. All authors read and approved the final manuscript.

## Conflict of Interest

JL is employed by Dalian Sunasia Tourism Holding Co., Ltd. SL is employed by Qingdao Polar Haichang Ocean Park. The remaining authors declare that the research was conducted in the absence of any commercial or financial relationships that could be construed as a potential conflict of interest.

## Publisher’s Note

All claims expressed in this article are solely those of the authors and do not necessarily represent those of their affiliated organizations, or those of the publisher, the editors and the reviewers. Any product that may be evaluated in this article, or claim that may be made by its manufacturer, is not guaranteed or endorsed by the publisher.

## Footnotes

1
https://software-ab.informatik.uni-tuebingen.de/download/megan6/welcome.html

2
https://www.megasoftware.net/

3
http://www.ebi.ac.uk/services/proteins

4
https://blast.ncbi.nlm.nih.gov

5
http://www.ebi.ac.uk/Tools/

6
https://rdrr.io/bioc/muscle/man/muscle-package.html

7
https://blast.ncbi.nlm.nih.gov/Blast.cgi

8
https://sray.med.som.jhmi.edu/SCRoftware/SimPlot/

## References

[B1] Appleton H. Higgins P. G. (1975). Letter: viruses and gastroenteritis in infants. *Lancet* 1:1297. 10.1016/s0140-6736(75)92581-7 48925

[B2] Boros A. Albert M. Pankovics P. Bíró H. Pesavento P. A. Phan T. G. (2017). Outbreaks of neuroinvasive astrovirus associated with encephalomyelitis, weakness, and paralysis among weaned pigs, hungary. *Emerg. Infect. Dis.* 23 1982–1993. 10.3201/eid2312.170804 29148391 PMC5708238

[B3] Calistri A. Palu G. (2015). Editorial commentary: unbiased next-generation sequencing and new pathogen discovery: undeniable advantages and still-existing drawbacks. *Clin. Infect. Dis.* 60 889–891. 10.1093/cid/ciu913 25572900

[B4] Cortez V. Margolis E. Schultz-Cherry S. (2019). Astrovirus and the microbiome. *Curr. Opin. Virol.* 37 10–15. 10.1016/j.coviro.2019.05.002 31163291 PMC6768711

[B5] Finkbeiner S. R. Allred A. F. Tarr P. I. Klein E. J. Kirkwood C. D. Wang D. (2008). Metagenomic analysis of human diarrhea: viral detection and discovery. *PLoS Pathog.* 4:e1000011. 10.1371/journal.ppat.1000011 18398449 PMC2290972

[B6] Finkbeiner S. R. Li Y. Ruone S. Conrardy C. Gregoricus N. Toney D. (2009). Identification of a novel astrovirus (astrovirus VA1) associated with an outbreak of acute gastroenteritis. *J. Virol.* 83 10836–10839. 10.1128/JVI.00998-09 19706703 PMC2753140

[B7] Johnson C. Hargest V. Cortez V. Meliopoulos V. A. Schultz-Cherry S. (2017). Astrovirus pathogenesis. *Viruses* 9:22. 10.3390/v9010022 28117758 PMC5294991

[B8] Karlsson E. A. Small C. T. Freiden P. Feeroz M. M. Matsen F. A. San S. (2015). Non-human primates harbor diverse mammalian and avian astroviruses including those associated with human infections. *PLoS Pathog.* 11:e1005225. 10.1371/journal.ppat.1005225 26571270 PMC4646697

[B9] Meliopoulos V. A. Kayali G. Burnham A. Oshansky C. M. Thomas P. G. Gray G. C. (2014). Detection of antibodies against Turkey astrovirus in humans. *PLoS One* 9:e96934. 10.1371/journal.pone.0096934 24826893 PMC4020816

[B10] Mitchell D. K. Matson D. O. Cubitt W. D. Jackson L. J. Willcocks M. M. Pickering L. K. (1999). Prevalence of antibodies to astrovirus types 1 and 3 in children and adolescents in Norfolk. Virginia. *Pediatr. Infect. Dis. J.* 18 249–254. 10.1097/00006454-199903000-00008 10093946

[B11] Monroe S. S. Carter M. J. Hermann J. Mitchell D. K. Sanchez-Fauquier A. (2005). “Astroviridae,”. in *Virus Taxonomy: Eighth Report of the International Committee on Taxonomy of Viruses*, eds Fauquet C. M. Mayo M. A. Maniloff J. Desselberger U. Ball L. A. Amsterdam (San Diego: Academic Press), 859–864.

[B12] Parry R. Asgari S. (2019). Discovery of novel crustacean and cephalopod flaviviruses: insights into the evolution and circulation of flaviviruses between marine invertebrate and vertebrate hosts. *J. Virol.* 93:e00432-19. 10.1128/JVI.00432-19 31068424 PMC6600200

[B13] Rivera R. Nollens H. H. Venn-Watson S. Gulland F. M. Wellehan J. F. (2010). Characterization of phylogenetically diverse astroviruses of marine mammals. *J. Gen. Virol.* 91 166–173. 10.1099/vir.0.015222-0 19759240

[B14] Roach S. N. Langlois R. A. (2021). Intra- and cross-species transmission of astroviruses. *Viruses* 13:1127. 10.3390/v13061127 34208242 PMC8230745

[B15] Sadeghi M. Altan E. Deng X. Barker C. M. Fang Y. Coffey L. L. (2018). Virome of > 12 thousand *Culex* mosquitoes from throughout California. *Virology* 523 74–88. 10.1016/j.virol.2018.07.029 30098450

[B16] Tamura K. Stecher G. Peterson D. Filipski A. Kumar S. (2013). MEGA6: molecular evolutionary genetics analysis version 6.0. *Mol. Biol. Evol.* 30 2725–2729. 10.1093/molbev/mst197 24132122 PMC3840312

[B17] Vu D. L. Bosch A. Pinto R. M. Guix S. (2017). Epidemiology of classic and novel human astrovirus: gastroenteritis and beyond. *Viruses* 9:33. 10.3390/v9020033 28218712 PMC5332952

[B18] Walter J. E. Mitchell D. K. (2000). Role of astroviruses in childhood diarrhea. *Curr. Opin. Pediatr.* 12 275–279. 10.1097/00008480-200006000-00018 10836166

[B19] Wu Z. Lu L. Du J. Yang L. Ren X. Liu B. (2018). Comparative analysis of rodent and small mammal viromes to better understand the wildlife origin of emerging infectious diseases. *Microbiome* 6:178. 10.1186/s40168-018-0554-9 30285857 PMC6171170

[B20] Yang J. Yang F. Ren L. Xiong Z. Wu Z. Dong J. (2011). Unbiased parallel detection of viral pathogens in clinical samples by use of a metagenomic approach. *J. Clin. Microbiol.* 49 3463–3469. 10.1128/JCM.00273-11 21813714 PMC3187305

